# Movement restrictions, vaccine coverage, and reduction of the COVID-19 incidence rate in the fourth wave of the pandemic: Analysis results from 63 provinces in Vietnam

**DOI:** 10.3389/fpubh.2022.988107

**Published:** 2023-01-12

**Authors:** Hanh My Bui, Minh Hoang Ha, Thang Phuoc Dao, Manh Duy Vu, Thai Quang Pham, Minh Loi Nguyen, Minh Hong Phan, Mai Thi Thanh Nguyen, Xuyen Hong Thi Hoang, Huong Thu Thi Ngo, Minh Do Van, Cuong Le Quang

**Affiliations:** ^1^Department of Tuberculosis and Lung Disease, Hanoi Medical University, Hanoi, Vietnam; ^2^Center for Development of Curriculum and Human Resources in Health Hanoi Medical University, Hanoi, Vietnam; ^3^Department of Functional Exploration, Hanoi Medical University Hospital, Hanoi, Vietnam; ^4^Department of Scientific Research and International Cooperation, Hanoi Medical University Hospital, Hanoi, Vietnam; ^5^ORLab, Faculty of Computer Science, Phenikaa University, Hanoi, Vietnam; ^6^Department of Communicable Diseases Control and Prevention, National Institute of Hygiene and Epidemiology, Hanoi, Vietnam; ^7^Administration of Science Technology and Training, Ministry of Health Vietnam, Hanoi, Vietnam; ^8^Outpatients Department, Bach Mai Hospital, Hanoi, Vietnam; ^9^Department of Pediatrics, Hanoi Medical University, Hanoi, Vietnam; ^10^Department of Orthopedic, Hanoi Medical University, Hanoi, Vietnam; ^11^Department of Neurology, Hanoi Medical University, Hanoi, Vietnam

**Keywords:** COVID-19, movement restrictions, vaccine coverage, Vietnam, fourth wave

## Abstract

On April 27, 2021, the fourth wave of the coronavirus disease 2019 (COVID-19) pandemic originating from the Delta variant of the severe acute respiratory syndrome coronavirus 2 (SARS-CoV-2) began in Vietnam. The adoption of travel restrictions, coupled with rapid vaccination and mask-wearing, is a global strategy to prevent the spread of COVID-19. Although trade-off between health and economic development are unavoidable in this situation, little evidence that is specific to Vietnam in terms of movement restrictions, vaccine coverage, and real-time COVID-19 cases is available. Our research question is whether travel restrictions and vaccine coverage are related to changes in the incidence of COVID-19 in each province in Vietnam. We used Google's Global Mobility Data Source, which reports different mobility types, along with reports of vaccine coverage and COVID-19 cases retrieved from publicly and freely available datasets, for this research. Starting from the 50th case per province and incorporating a 14-day period to account for exposure and illness, we examined the association between changes in mobility (from day 27 to 04–03/11/2021) and the ratio of the number of new confirmed cases on a given day to the total number of cases in the past 14 days of indexing (the potentially contagious group in the population) per million population by making use of LOESS regression and logit regression. In two-thirds of the surveyed provinces, a reduction of up to 40% in commuting movement (to the workplace, transit stations, grocery stores, and entertainment venues) was related to a reduction in the number of cases, especially in the early stages of the pandemic. Once both movement and disease prevalence had been mitigated, further restrictions offered little additional benefit. These results indicate the importance of early and decisive actions during the pandemic.

## Introduction

The governments of all countries worldwide have mobilized their resources to control the COVID-19 pandemic. A review conducted by Oh et al. (1) in 34 countries showed that non-pharmacological interventions (NPIs) such as the closure of schools and universities are highly effective; restricting gatherings and the closure of high-risk businesses are effective but closing most other businesses is less beneficial; and many countries can reduce R to below 1 without issuing stay-at-home orders and imposing drastic sanctions such as limitations on travel and the closure of non-essential businesses. Recent analyzes suggest that these large-scale NPIs are jointly effective in reducing baseline infection rates to <1 when applied concurrently. Movement restrictions are one of eight NPIs used in the response to the COVID-19 pandemic in most provinces and include working from home (facilitated by the expansion of resources that enable online meetings, teaching, and shopping), stay-at-home orders, closing shops and offices, and stopping public transport (2–5). These measures have been implemented in many countries, including Vietnam, in response to the fourth wave of the COVID-19 pandemic (6–8).

The rationale for restricting movement is that limiting personal and social activities will reduce the amount, duration, and extent of close interpersonal contact—the source of the virus infection—like simulations using a complex mathematical model of the 1918 influenza epidemic (9), as well as assumptions about the current size and nature of exposure in the population (10). These initial models depended on an understanding of airborne, droplet, and contact transmission mechanisms, leading to the development of experimental, and evidence-based models. Behavioral and social change in terms of reducing interpersonal contact and increasing distance will reduce the spread of respiratory infections (11). Nevertheless, the paucity of studies on nationwide mobility restrictions in LMIC countries such as Vietnam during Delta variant outbreaks has limited the scope of research on their impact on transmission. In spite of this gap in knowledge, the rapid progression of this pandemic has forced many governments to experiment with different prevention methods with limited evidence of their effectiveness (12).

More common public health prevention strategies (including the quarantining of contacts, isolation of infected people, and contact tracing) and control measures within the health system (such as patient flow segregation and the use of negative pressure ventilation and personal protective equipment) have largely been applied for pandemic control in many countries, including Vietnam (13–16). Mobility restriction is considered a non-pharmacological approach to large-scale behavioral and social interventions with a considerable outcome in terms of cost but no option (14, 17). Estimates suggest that global GDP growth has fallen by as much as 10% (18), at least part of which may be due to these mobility limitations. Although points of view differ, especially given the relative lack of information on what would happen if the disease was not brought under control [like the dire situation in India at the beginning of 2021, which is a profound lesson in inadequate initial recognition of the condition leading to persisting disability in a significant number of survivors (long COVID)], at the start of the pandemic, some voiced concerns that the economic damage caused by restrictive measures would be greater than that accounted for by the direct impact of COVID-19 morbidity and mortality (19, 20).

Some studies have begun to find an association between movement limitations and decreased transmission of the virus (21, 22), while a recent analysis on the impact of COVID-19 demonstrated that inadequate pandemic control capacity, from communities to health facilities, tends to be related to economic loss, and quality of life (23). As the pandemic has entered its fifth wave, governments, and supporters of the rapid easing of restrictions have no choice but to face the risk of economic exhaustion. This study is the first to provide a complete analysis of infection incidence, mobility, and vaccine coverage by assessing the association between mobility patterns and the risk of COVID-19 in 63 provinces and cities in Vietnam.

## Methods

### Population and data

The study population included the populations of 63 provinces and cities in Vietnam. We collected the daily records of new COVID-19 cases and confirmed deaths by province from a dataset publicly available at https://covid.ncsc.gov.vn/. The dataset is used as the official COVID-19 data page of the Vietnam government which has been operated by the National Technology Center for COVID-19 prevention. The data included on the website have been collected from various information sources while most of them were exported from the health registries. During the studied period, people infected with COVID-19 were required either to complete an online medical declaration or to seek the treatment services. The COVID-19 patients were granted many benefits such as social insurance, free treatment, isolation along with routine care, and support for necessity. Along with the extensive anti-epidemic propaganda by the government as well as the self-consciousness of the people, the number of undeclared people remained minor. The fact is that, many Vietnamese people completed the self-testing at home even though they were asymptomatic, by the time their family members had got infected, and then reported the testing results to the local commune health cancer. In addition, the province-based mobility data were obtained from Google's Global Mobility Data Source (24). The data was derived from mobile based global positioning system (GPS) information from people who agreed to share the anonymized location data with Google. The data were classified by Google into six different types of places visited (workplaces, transit stations, retail stores, entertainment venues, residential areas, grocery stores, drugstores, and parks). Finally, we collected data following the number of vaccinations per province as well as the population statistics at provincial level from the report of the Central Institute of Hygiene and Epidemiology. It is noted that the dataset included both symptomatic and asymptomatic COVID-19 patients.

### Parameters and variables

The parameters and variables analyzed based on the six types of sites included:

- *MR*_*i*_: The value of commuting mobility on day *i*, calculated as the average of the five values of workplaces, grocery stores and pharmacies, retail shops and entertainment venues, transit stations, and parks. Compared with study of Oh et al. (1), due to the customary characteristics as well as the content of the social distancing order, we added grocery stores, pharmacies, and parks to the calculation. Although grocery stores and pharmacies are considered essential, due to the movement restrictions in Vietnam, these two values also reduced and were close to other mobility values.
- *c*_*i*_: Number of new confirmed COVID-19 cases on day *i*.
- *v*_*i*_*:* The proportion of people who received at least one dose of the vaccine by day *i*. Because the number of people who received two doses of the vaccine as of October 20 was still low, we only evaluated the effectiveness of the vaccine in the model through the percentage of people who received at least one vaccine dose (regardless of type).
- *GR*_*i*_: Represents the rate of spread of the disease on day *i*. Based on study of Oh et al. (1), we built on GR_i_ according to the following formula:

$$\begin{array}{l} R_{i\;}=\frac{\overset{6}{\underset{k\;=\;0}{\sum }}\frac{c_{i\;-\;k}}{7}}{\overset{13}{\underset{l\;=\;0}{\sum }}\frac{c_{i\;-\;l}}{14}} \end{array}$$

The value and meaning of the GR_i_ variable are provided and explained in study of Oh et al. (1). The number of days counted was doubled because COVID-19 now has more new variants, leading to a larger time range in which patients are identified as being infected as well as data entry time in each area. After many factor tests, 7 and 14 days were chosen as they gave the best results.

### Analysis

Similar to study of Oh et al. (1), we applied a generalized linear model (GLM) to each area starting from the 50th case detected. The GLM model from MR_i_ to GR_i_ was tested over different lag-day values from 9 to 24 days, following which the best lag-day value for all regions was selected. Regarding regions, we conducted an overall analysis on the whole of Vietnam, region by region, and finally on the 18 provinces with the leading number of cases (>6,000 cases; two provinces, Kien Giang and Ca Mau, were excluded due to numerous data entry errors).

MR_i_ was specifically applied during the reference period (April 27–October 20, 2021, the time period during which experts from India were identified when entering and working in Vietnam and COVID-19 was declared a global pandemic, and the earliest time period for which data were available) as the basis of the calculated changes.

The Pearson correlation coefficient (*r*) was used to evaluate the experiment as well as the slope. We defined the correlation between the measures based on the *r* value as follows:

- +0.70 or higher: Very strong positive relationship.
- +0.40 to +0.69: Strong positive relationship.
- +0.30 to +0.39: Moderate positive relationship.
- +0.20 to +0.29: Weak positive relationship.
- +0.01 to +0.19: No or negligible relationship.
- 0: No relationship (zero correlation).
−0.01 to −0.19: No or negligible relationship.
−0.20 to −0.29: Weak negative relationship.
−0.30 to −0.39: Moderate negative relationship.
−0.40 to −0.69: Strong negative relationship.
−0.70 or higher: Very strong negative relationship.

Finally, based on the results obtained from the above model, we continued to build a linear model applying more vaccine data. Because the number of people who received the second dose of vaccine was small, we only used data on the proportion of people who received at least one dose of vaccine per population in each region (v_i_). We added the constraint that the coefficient (slope) of GR_i_ must not be negative and the coefficient of v_i_ must not be positive, with the expectation that MR_i_ is proportional to GR_i_ and vice versa to v_i_ and that the value of v_i_ is inversely proportional to GR_i_. V_i_ data were also applied to lag-day (vaccine lag-day) compared with MR_i_ to ensure that the body produces heterogeneity after injection. For example, for a lag-day vaccine value of 14, the MR_i_ was combined with the micro-14 vaccine data. We also examined lag-day vaccines from 7 to 30 days and selected the best lag-day vaccine for the region.

## Results

In this section, the obtained results are summarized and analyzed. We note that the detailed results can be found in Appendix. Table 1 reports the total number of COVID-19 cases and the ratio of infected cases in the population across all 63 assessed provinces. As of November 3, 2021, the total number of cases in Vietnam was 1,144,374 cases, mainly concentrated in the southern provinces, which accounted for 1,064,389 cases as opposed to the relatively low number of cases in the northern and central provinces (17,420 and 43,417 cases, respectively).

**Table 1:** Total number of COVID-19 cases the ratio of infected cases in the population from 27/4/2021 to 13/11/2021.

**Province**	**Cases**	**Ratio (%)**	**Province**	**Cases**	**Ratio (%)**	**Province**	**Cases**	**Ratio (%)**
Ho Chi Minh City	443,409	4.7112	Tra Vinh	4,029	0.3988	Nam Dinh	688	0.0388
Binh Duong	242,735	9.0633	Phu Yen	3,264	0.3730	Quang Tri	588	0.0920
Ca Mau	105,316	8.8352	Ben Tre	3,264	0.2520	Hung Yen	523	0.0409
Dong Nai	77,417	2.3922	Ninh Thuan	3,033	0.5092	Hai Duong	385	0.0199
Kien Giang	46,790	2.7044	Nghe An	2,996	0.0877	Vinh Phuc	339	0.0286
Long An	36,438	2.0892	Binh Phuoc	2,858	0.2800	Kon Tum	335	0.0592
Tien Giang	20,071	1.1256	Bac Ninh	2,681	0.1848	Son La	327	0.0254
An Giang	16,764	0.8990	Gia Lai	2,293	0.1463	Thai Binh	276	0.0147
Tay Ninh	15,656	1.3147	Binh Dinh	2,266	0.1524	Lang Son	269	0.0340
Dong Thap	13,319	0.8396	Ha Giang	2,176	0.2463	Quang Ninh	231	0.0170
Khanh Hoa	10,081	0.8088	Quang Binh	2,123	0.2344	Dien Bien	219	0.0351
Can Tho	9,150	0.7351	Hau Giang	2,053	0.2819	Ninh Binh	192	0.0192
Binh Thuan	8,412	0.6762	Quang Ngai	1,975	0.1600	Lao Cai	146	0.0193
Soc Trang	7,527	0.6369	Quang Nam	1,713	0.1134	Hai Phong	143	0.0069
Bac Lieu	6,608	0.7200	Thua Thien Hue	1,576	0.1386	Thai Nguyen	84	0.0064
Ba Ria–Vung Tau	6,549	0.5544	Thanh Hoa	1,503	0.0407	Tuyen Quang	46	0.0058
Bac Giang	6,392	0.3439	Phu Tho	1,401	0.0937	Lai Chau	35	0.0046
Ha Noi	6,141	0.0729	Dak Nong	1,355	0.2076	Hoa Binh	32	0.0037
Dak Lak	5,708	0.3008	Ha Nam	1,190	0.1372	Yen Bai	19	0.0023
Da Nang	5,234	0.0043	Lam Dong	872	0.0660	Bac Kan	11	0.0035
Vinh Long	4,436	0.4393	Ha Tinh	704	0.0541	Cao Bang	8	0.0015
Northern Vietnam	17,420							
Central Vietnam	43,417							
Southern Vietnam	1,064,389							
Vietnam	1,144,374							

The effect of movement limitations on the number of cases was calculated through the variation of MR_i_ across regions. The results are presented in Table 2. In general, from the 50th case since April 27, 2021, the MR_i_ tended to decrease right after the outbreak; thereafter, when the epidemic situation showed signs of infection control, the MR_i_ was also restored. Table 2 presents the MR_i_ change data: the “Province” column shows the region name, the “Before” column shows the highest MR_i_ value before movement restrictions from the 50th case date, the “Min” and “Date of Min” columns indicate the smallest MR_i_ value corresponding to the recorded date, the “Last” column (October 20) shows the last value of the MR_i_ on October 20, 2021, the “Decrease” column shows the difference between Before and Min, and the “Recover” column indicates the difference between Last and Min.

**Table 2:** Variation of MR_i_ by province.

**Province**	**Before**	**Min**	**Date of Min**	**Last (20/10)**	**Decrease**	**Recover**
Ho Chi Minh City	−19.800	−94.400	23/08	−48.600	74.600	45.800
Binh Duong	−4.000	−83.400	23/08	−36.200	79.400	47.200
Dong Nai	−13.000	−81.200	28/08	−37.600	68.200	43.600
Long An	−16.600	−80.000	28/08	−17.800	63.400	62.200
Tien Giang	−46.200	−83.400	28/08	−56.200	37.200	27.200
An Giang	−17.000	−73.400	22/08	−38.400	56.400	35.000
Tay Ninh	−21.400	−86.400	19/08	−36.800	65.000	49.600
Dong Thap	−28.200	−79.200	22/08	−36.200	51.000	43.000
Khanh Hoa	−33.200	−85.000	15/08	−43.000	51.800	42.000
Can Tho	−55.600	−81.200	25/08	−33.200	25.600	48.000
Binh Thuan	−43.600	−70.200	03/09	−36.600	26.600	33.600
Soc Trang	−66.200	−72.800	07/08	−31.600	6.600	41.200
Bac Lieu	−58.600	−76.400	28/08	−22.600	17.800	53.800
Ba Ria–Vung Tau	−17.400	−82.600	03/09	−28.200	65.200	54.400
Bac Giang	−29.800	−60.600	27/05	26.800	30.800	87.400
Ha Noi	−17.000	−81.400	02/09	−22.400	64.400	59.000
Northern Vietnam	−13.744	−30.696	13/05	4.776	16.952	35.472
Central Vietnam	−13.379	−62.295	02/09	−20.884	48.916	41.411
Southern Vietnam	3.274	−68.968	28/08	−28.337	72.242	40.631
Vietnam	19.159	−50.883	03/09	−12.949	70.042	37.934

Regarding the GLM model, we found the best lag-day value from MR_i_ to GR_i_ for all regions. Values were tested from 9 to 24 days. Table 3 shows that the mean of r as a lag-day value takes the form of an ascending mid-range, peaking at 0.498 at a lag-day of 21 days. Thus, we chose a lag-day value of 21 for all regions.

**Table 3:** *r*-values according to mobility lag-day.

**Lag (days)**	**9**	**10**	**11**	**12**	**13**	**14**	**15**	**16**
AVG	0.311	0.341	0.360	0.376	0.391	0.409	0.431	0.445
MAX	0.580	0.604	0.650	0.686	0.712	0.720	0.734	0.751
MIN	0.039	0.066	0.050	0.037	0.015	−0.005	0.064	0.106
**Lag (days)**	**17**	**18**	**19**	**20**	**21**	**22**	**23**	**24**
AVG	0.458	0.477	0.489	0.497	0.498	0.493	0.491	0.484
MAX	0.758	0.766	0.768	0.765	0.755	0.725	0.744	0.740
MIN	0.124	0.216	0.246	0.260	0.268	0.232	0.182	0.155

**Figure 1:**
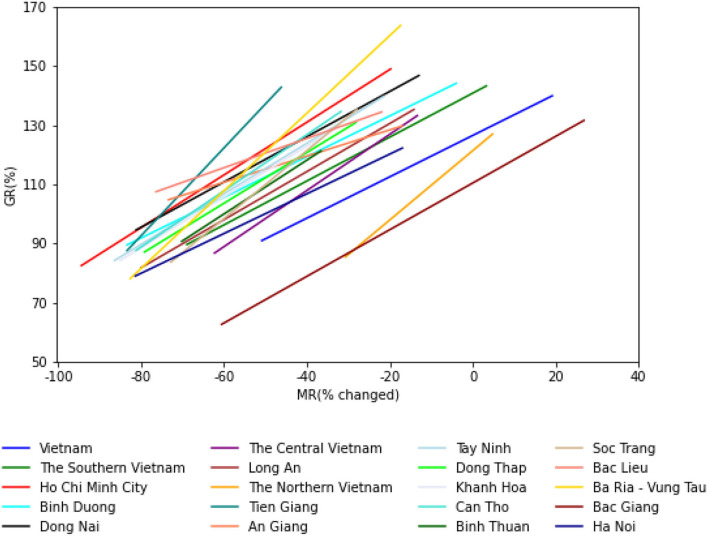
GR and MR rates across the country.

**Figure 2:**
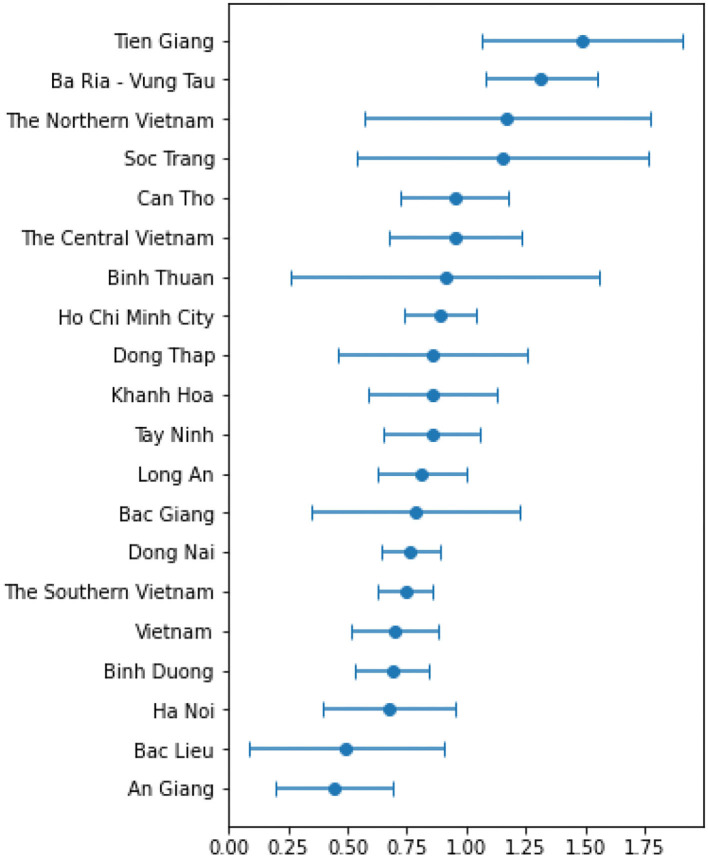
Slope results of MR_i_.

Figures 1, 2 and Table 4 present the results of GLM achieved with 21 days of mobility lag-day. Table 4 presents results of the linear mobility model with 21 lag-days per month. While Figure 1 visualizes the GR and MR rates across provinces in Vietnam, Figure 2 shows the 95% CI values for the estimated slope values. As expected, the results showed that in Vietnam, MR_i_ has a strong positive correlation with GR_i_ with an *r*-value of 0.496, slope 0.7. In three regions, r was proportional to the total number of cases, with the highest r value reaching 0.708 (very strong positive correlation), followed by the central region (0.462; strong positive correlation) and the north (0.285; weak positive correlation). However, the slope was in the reverse order because the mobility change was less in the north than in the central and southern regions. Thus, the larger the number of cases, the more the mobility restrictions implemented.

**Table 4:** Results of the linear mobility model with 21 lag-days per month.

**Provinces**	***r***	**Intercept**	**Slope**
Dong Nai	0.755	1.568	0.766
Ho Chi Minh City	0.695	1.667	0.892
Ba Ria–Vung Tau	0.695	1.867	1.314
Can Tho	0.641	1.649	0.951
Long An	0.627	1.469	0.814
Binh Duong	0.614	1.470	0.689
Tay Ninh	0.605	1.581	0.855
Tien Giang	0.543	2.116	1.487
Khanh Hoa	0.512	1.573	0.859
Dong Thap	0.379	1.552	0.860
Soc Trang	0.359	1.675	1.151
Ha Noi	0.345	1.337	0.672
An Giang	0.323	1.373	0.443
Binh Thuan	0.274	1.547	0.911
Bac Giang	0.268	1.105	0.790
Bac Lieu	0.266	1.454	0.496
Northern Vietnam	0.285	1.214	1.172
Central Vietnam	0.462	1.460	0.951
Southern Vietnam	0.708	1.409	0.743
Vietnam	0.496	1.266	0.700

Considering the provinces with a large number of cases, all provinces had r values >0.2 (positive relationship), and provinces with a large number of cases often had higher r values. Dong Nai was the province with the highest r value (0.755; very strong positive relationship). Eight provinces had *r*-values between 0.512 and 0.695 (strong positive relationship). Four provinces had r values ranging from 0.323 to 0.379 (moderate positive relationship). Only three provinces had r values in the range of 0.266–0.274 (weak positive relationship). These provinces had fewer cases compared to the other provinces considered; in addition, Bac Giang province controlled the epidemic very early (since the beginning of July), while the review period ended on October 20, 2021.

As the slope result of MR_i_ was always positive in the provinces considered (Figure 2), as mentioned above, when applying more vaccine data, we added the constraint that the slope of the GR_i_ should not be negative and the coefficient of v_i_ must not be positive. After surveying the lag-day vaccine from 7 to 30 days, we chose the lag-day vaccine with a value of 16 days, which yielded the highest average correlation coefficient of 0.532. Table 5 shows the results of applying the vaccine to the forced model. Column “*r*” shows the correlation coefficient of the first model, column “*r*′” indicates the correlation coefficient when applying the vaccine, column “S_m_” represents the slope coefficient of mobility, and column “S_v_” indicates the slope coefficient of the vaccine. After applying more vaccines, 12 regions out of 20 showed an *r*′ value greater than the *r*. Of these, four provinces had r' over 0.702 (very strong positive relationship). The regions that showed no change in the model after applying the vaccine (no impact of the vaccine) due to the recent increase in the number of cases may be facing the fifth wave of the epidemic.

**Table 5:** Results of the linear mobility model combining vaccine with 21 lag-days mobility and 16 lag-days vaccine.

**Province**	***r***	***r^′^***	**s_m_**	**s_v_**
Ho Chi Minh City	0.695	0.839	0.654	−0.393
Dong Nai	0.755	0.805	0.798	−0.236
Long An	0.627	0.750	1.040	−0.426
Binh Duong	0.614	0.702	0.664	−0.326
Ba Ria–Vung Tau	0.695	0.695	1.314	0
Can Tho	0.641	0.641	0.951	0
Tay Ninh	0.605	0.630	0.905	−0.649
Khanh Hoa	0.512	0.589	1.164	−0.399
Tien Giang	0.543	0.545	1.488	−0.134
Bac Lieu	0.266	0.423	1.152	−3.090
Dong Thap	0.379	0.379	0.860	0
An Giang	0.323	0.361	0.484	−0.676
Soc Trang	0.359	0.359	1.151	0
Ha Noi	0.345	0.345	0.672	0
Binh Thuan	0.274	0.274	0.911	0
Bac Giang	0.268	0.268	0.842	−0.188
Northern Vietnam	0.285	0.285	1.172	0
Central Vietnam	0.462	0.462	0.951	0
Southern Vietnam	0.708	0.756	0.615	−0.454
Vietnam	0.496	0.534	0.610	−0.370

## Discussion

### Mobility restrictions and number of cases

After assessing the detailed map of the regions in the Appendix, we noticed the following problems. Mobility restrictions were in place across all provinces in Vietnam. Before the implementation of mobility restrictions in each province, the level of mobility suddenly increased, especially in places like groceries and pharmacies, because people were worried and went out to buy food and medicine. Immediately afterwards (4–14 days), a very high number of new cases per day appeared, often forming a peak. During the initial period of implementation of restrictions, the MR_i_ value immediately decreased, leading to a decrease in GR_i_ along with the MR_i_, which was proportionally high at the uncontrolled stage of the epidemic, usually in the middle months, and low in the early stages and months. epidemic, the final stage when the epidemic is under control. At the end of the period, the epidemic situation seemed to be under control, with the MR_i_ value gradually increasing and the number of cases decreasing. However, in many regions, MR_i_ increased rapidly, which caused GR_i_ to increase again, leading to the fifth outbreak.

Our results were similar to the findings of other studies that evaluated the implementation of social distancing policies in the community (25, 26). The positive association between mobility and transmission rate was declared by Nouvellet et al. (25) using the data collected from 52 countries. The decline in community-based transmission rate was observed according to the duration of strict implementation of social distancing strategies (25). The surveillance data were collected from databases managed by WHO and European Centres for Disease Control, while the mobility was exported from the Google online database (25). Using the Google mobility data, Gregory et al. concluded that social distancing strategies significantly promoted the decrease in the number of new COVID-19 cases detected in the community (26). Wellenius et al. (26) also considered that the risk of pandemic re-emergence might be available in the context of school and community re-opening. In the study conducted by Courtemanche et al. (27) the pandemic re-emergence was also predicted to occur in the community after the 2-month duration of reopening schools in Texas, which might increase the mortality rate.

Strict measures to limit movement and support for digital interventions such as tracing, reporting, and mobility proved effective at the beginning of the pandemic and are evident in the low basic infection rates in Vietnam. The local government strictly implemented the strategies of case investigation and contact tracing, followed by routine health declaration activities. Subsequently, Vietnam witnessed a specific time when new cases with positive testing results were not detected in the community and the routine activities of the residents living in areas where the series of COVID-19 patients had been seen previously returned to normal. During the recovery period, the approaches of restricting movement were eased while the case surveillance activities were still strictly maintained in the community, especially the immigration controls at boundary areas and airports. Since the first wave of the pandemic, the number of infected cases surged and reached its peak during August and September 2021. To minimize the community-based transmission, the methods of restricting movements and isolating were extremely re-implemented across the epidemiological hotspots whereas contact tracing was not prioritized due to the wide distribution of the infection source.

We observed that the changes in mobility across provinces declined not drastically before the most severe duration of the pandemic outbreak, from April to June in 2022, accompanied by the coverage of vaccination campaigns not being as expected. Nevertheless, from that point, community-based mobility started significantly changing with the downward trends predominantly recorded during the study time. The mobility in Ho Chi Minh City, the center of the pandemic in Vietnam in 2022, presented an upward trend in areas of groceries and pharmacies as well as the transit stations during the pre-peak duration of the pandemic. The residents attempted to visit groceries and pharmacies to purchase food and medicines as the backup in the context that supply chains would be locally restricted to adapt to COVID-19 prevention. Moreover, the considerable number of immigrants moved to their hometowns to avoid the infection caused an increase in mobility at transit stations, which returned to increase as the immigrants moving back to the city for study and work after the pandemic subsided. The close contacts, who are called F1 and defined as having close contact with infected cases at a distance of under 2 m and time of over 3 min, were isolated and suffering close health monitoring at the quarantined areas.

However, restricting movement and isolating, tracing, and zoning out all those considered to be in contact with COVID-19 patients consumes considerable economic and human resources and time. A significant number of healthcare workers were delivered to the front line to support the prevention activities which cause the shortage of manpower to maintain the routine surveillance of other seasonal infectious diseases (e.g., dengue and influenza). Furthermore, the movement restriction additionally might cause the risk of occurring an economic crisis reflected through the collapse in mobility to the areas of retails, recreation, and workplaces. The less frequently the business activities happen, the more possibly the economic crisis might occur which negatively affects the low-middle income households in the epidemical hotspots. If other NPIs such as face coverings and social distancing are strictly followed, the localized and isolated outbreaks in Ho Chi Minh City could be prevented and the goals of eliminating pandemic in other neighboring provinces (e.g., Dong Nai, Binh Duong) will be observed and feasible. Mobility restrictions may be still required again, but only as a measure of last resort and considered likely evidence when the policy fails.

### Effect of number of cases and vaccine coverage rate

With the efforts of controlling the pandemic, vaccination campaigns were widely organized in the community which were reflected by the dramatic increase in number of vaccinated cases since July 2021. There were eight types of vaccines were officially approved by the Ministry of Health and widely used in Vietnam during the considered period, including: (1) AZD1222, AstraZeneca (approved on January 1, 2021), (2) Sputnik-V, Jambalaya (approved on March 3, 2021), (3) Vero-Cell, Sinopharm (approved on June 3, 2021), (4) Comirnaty, Pfizer BioNtech (approved on June 16, 2021), (5) Spikevax, Moderna (approved on June 28, 2021), (6) Janssen, Janssen Pharmaceutica NV and Janssen Biologics B.V (approved on July 15, 2021), (7) Hayat-Vax, China National Biotec Group (approved on September 10, 2021), and (8) Abdala, AICA Laboraties—Base Business Unit (BBU) AICA—Cuba (approved on September 17, 2021) (28). The enhanced coverage of vaccination campaigns strongly correlated with the decline in the number of new COVID-19 in the locations of pandemic centers. After the vaccination rate increased, the number of deaths tended to decrease steadily. The number of deaths has decreased significantly, although the number of cases is still increasing. Expected results of the implementation of restrictive travel policies as well as rapid vaccination coverage associated with a reduction in the occurrence of new COVID-19 cases were confirmed in this and many similar studies, even in pre-vaccination periods, with a minimum reduction of 20–40%. Mortality mitigation is enhanced as vaccine coverage gradually increases and continues as low-level movement restrictions remain. Movement restrictions have the most impact on the number of cases in the early stages when the vaccination rate is not high, and this impact begins to decrease when social distancing is eased, although the rate of vaccine coverage continues to increase. These findings are consistent with other studies showing the benefits of early travel restrictions and vaccine coverage (29, 30). Previous studies of government-initiated interventions have demonstrated an association with reduced incidence of COVID-19, although they did not directly assess the magnitude of reduced mobility or the temporal impact of interventions.

## Limitations

The first limitation of our study on mobility and COVID-19 is that information was obtained from Google Mobile's internet data source based on Android phone users with location services enabled. This number of subjects may not be enough to represent all 63 provinces in Vietnam. In addition, data on case numbers may have been affected by availability, testing policy, and reporting quality. Finally, the use of this type of data can be confounded and biased by complicating factors that do not separate the impact of mobility restrictions from many other intervention variables such as the degree of heterogeneity between households, the ability to detect and control outbreaks by wearing masks and implementing regulations on maintain a physical distance of 2 meters.

In addition, these factors are clearly influenced by the community's awareness and beliefs as well as the need and motivation to live and continue with jobs that are being severely affected by the pandemic. One possible limitation is that the relationships among actors involved in local policy implementation are not simple linear relationships and cannot be controlled by any intelligent algorithm.

Moreover, our study only used data from May 3, 2021, to November 3, 2021, when a series of localities were freed from movement restrictions, and the level of vaccine coverage was also taken into account. As a result, the number of deaths decreased markedly even though the number of cases per day continued to increase due to the simultaneous start of the fifth epidemic wave when implementing policy easing as well as the appearance of a new variant, Omicron. Therefore, further research is still necessary to study the effectiveness of a variety of mitigation measures as well as to improve methods of data collection and analysis regarding human behavior and infections.

Finally, the application of the GLM might result in potential disadvantages which showed that the linear correlation between variables was not established. Other methods, such as mutual information entropy which was applied by Lansiaux et al. (31), should be applied as an alternative. We would take into account the comparative evaluation of different NPIS assessment methods in our future research.

## Conclusion

Our study provides findings which strengthen the importance of social distancing strategies to minimize the risk of person-to-person transmission. The social distancing methods prove the significant contributions to COVID-19 prevention in the community. However, the gradual increase in the number of COVID-19 cases was observed during the early phase of the “New normal.” For this reason, community-based transmission should still be under routine surveillance to prevent any further risk of pandemic re-emergence. In addition, the effect of movement restrictions is evident, but it is crucial to calculate the effect of movement restrictions in response to COVID-19 and vaccine coverage with different socio-economic impacts.

## Data availability statement

The raw data supporting the conclusions of this article will be made available by the authors, without undue reservation.

## Author contributions

HB designed the study. HB and TP collected the data. MH and MV conducted data analysis. HB and MH wrote the manuscript. MVu, TP, MLN, MP, MTTN, XH, HN, MVa, TPD, and CQ reviewed the manuscript. All authors contributed to the article and approved the submitted version.

## References

[1] Oh J Lee H-Y Khuong QL Markuns JF Bullen C Barrios OEA. Mobility restrictions were associated with reductions in COVID-19 incidence early in the pandemic: evidence from a real-time evaluation in 34 countries. Sci Rep. (2021) 11:13717. 10.1038/s41598-021-92766-z 34215764 PMC8253807

[2] Rahman MM Thill J-C Paul KC. COVID-19 pandemic severity, lockdown regimes, and people's mobility: early evidence from 88 countries. Sustainability. (2020) 12:9101. 10.3390/su12219101

[3] Wei X Li L Zhang F. The impact of the COVID-19 pandemic on socio-economic and sustainability. Environ Sci Pollut Res. (2021) 28:68251–60. 10.1007/s11356-021-14986-0 34268692 PMC8282265

[4] Flaxman S Mishra S Gandy A Unwin HJT Mellan TA Coupland H. Estimating the effects of non-pharmaceutical interventions on COVID-19 in Europe. Nature. (2020) 584:257–61. 10.1038/s41586-020-2405-7 32512579

[5] Sandford A. Coronavirus: Half of Humanity Now on Lockdown as 90 Countries Call for Confinement. Euronews (2020). Available online at: https://www.euronews.com/2020/04/02/coronavirus-in-europe-spain-s-death-toll-hits-10-000-after-record-950-new-deaths-in-24-hou (accessed March 05, 2022).

[6] Brauner JM Mindermann S Sharma M Johnston D Salvatier J Gavenčiak T. The effectiveness of eight nonpharmaceutical interventions against COVID-19 in 41 countries. medRxiv. (2020). 10.1101/2020.05.28.20116129

[7] Liu H Bai X Shen H Pang X Liang Z Liu Y. Synchronized travel restrictions across cities can be effective in COVID-19 control. medRxiv. (2020). 10.1101/2020.04.02.20050781

[8] Manh TDN. We have Entered Phase 3 of the ‘War' Against COVID-19. Vu Du'c Dam. Available online at: http://vuducdam.chinhphu.vn/Home/Chung-ta-da-buoc-sang-giai-doan-3-cuoc-chien-chong-dich-COVID19/20204/24322.vgp (accessed March 05, 2022).

[9] Hiệp L. Overview of 3 Phases of COVID-19 Epidemic in Vietnam. Thanh Nien. Available online at: https://thanhnien.vn/thoi-su/toan-canh-3-giai-doan-dich-covid-19-tai-viet-nam-1207707.html (accessed March 05, 2022).

[10] Mwalili S Kimathi M Ojiambo V Gathungu D Mbogo R. SEIR model for COVID-19 dynamics incorporating the environment and social distancing. BMC Res Notes. (2020) 13:352. 10.1186/s13104-020-05192-1 32703315 PMC7376536

[11] Chu DK Akl EA Duda S Solo K Yaacoub S Schünemann HJ. Physical distancing, face masks, and eye protection to prevent person-to-person transmission of SARS-CoV-2 and COVID-19: a systematic review and meta-analysis. Lancet. (2020) 395:1973–87. 10.1016/S0140-6736(20)31142-9 32497510 PMC7263814

[12] Hsiang S Allen D Annan-Phan S Bell K Bolliger I Chong T. The effect of large-scale anti-contagion policies on the COVID-19 pandemic. Nature. (2020) 584:262–7. 10.1038/s41586-020-2404-8 32512578

[13] Wang Q Wang X Lin H. The role of triage in the prevention and control of COVID-19. Infect Control & Hosp Epidemiol. (2020) 41:772–6. 10.1017/ice.2020.185 32362296 PMC7231666

[14] Duong DM Le VT Ha BTT. Controlling the COVID-19 pandemic in vietnam: lessons from a limited resource country. Asia Pac J Public Health. (2020) 32:161–2. 10.1177/1010539520927290 32429676 PMC8685461

[15] Livingston E Desai A Berkwits M. Sourcing personal protective equipment during the COVID-19 pandemic. JAMA. (2020) 323:1912–4. 10.1001/jama.2020.5317 32221579

[16] Ayebare RR Flick R Okware S Bodo B Lamorde M. Adoption of COVID-19 triage strategies for low-income settings. Lancet Respir Med. (2020) 8:e22. 10.1016/S2213-2600(20)30114-4 32171063 PMC7103952

[17] Han E Tan MMJ Turk E Sridhar D Leung GM Shibuya K. Lessons learnt from easing COVID-19 restrictions: an analysis of countries and regions in Asia Pacific and Europe. Lancet. (2020) 396:1525–34. 10.1016/S0140-6736(20)32007-9 32979936 PMC7515628

[18] Griffin J Casey M Collins Á Hunt K McEvoy D Byrne A. Rapid review of available evidence on the serial interval and generation time of COVID-19. BMJ Open. (2020) 10:e040263. 10.1136/bmjopen-2020-040263 33234640 PMC7684810

[19] Syed AA Gupta S Rai D. Psychological, social and economic impact of COVID 19 on the working population of India: exploratory factor analysis approach. Int J Disaster Risk Reduct. (2021) 66:102617. 10.1016/j.ijdrr.2021.102617 34642625 PMC8494622

[20] Louhichi W Ftiti Z Ameur HB. Measuring the global economic impact of the coronavirus outbreak: evidence from the main cluster countries. Technol Forecast Soc Change. (2021) 167:120732. 10.1016/j.techfore.2021.120732 33723464 PMC7942192

[21] Kephart JL Delclòs-Alió X Rodríguez DA Sarmiento OL Barrientos-Gutiérrez T Ramirez-Zea M. The effect of population mobility on COVID-19 incidence in 314 Latin American cities: a longitudinal ecological study with mobile phone location data. Lancet Dig Health. (2021) 3:e716–22. 10.1016/S2589-7500(21)00174-6 34456179 PMC8545654

[22] Vo H-L Nguyen HAS Nguyen KN Nguyen HLT Nguyen HT Nguyen LH. Adherence to social distancing measures for controlling COVID-19 pandemic: successful lesson from Vietnam. Front Public Health. (2020) 8:589900. 10.3389/fpubh.2020.589900 33304878 PMC7701304

[23] Tran BX Nguyen HT Le HT Latkin CA Pham HQ Vu LG. Impact of COVID-19 on economic well-being and quality of life of the Vietnamese during the national social distancing. Front Psychol. (2020) 11:565153. 10.3389/fpsyg.2020.565153 33041928 PMC7518066

[24] Google. Google COVID-19 Community Mobility Reports. California City, CA: Google (2020).

[25] Nouvellet P Bhatia S Cori A Ainslie KEC Baguelin M Bhatt S. Reduction in mobility and COVID-19 transmission. Nat Commun. (2021) 12:1090. 10.1038/s41467-021-21358-2 33597546 PMC7889876

[26] Wellenius GA Vispute S Espinosa V Fabrikant A Tsai TC Hennessy J. Impacts of social distancing policies on mobility and COVID-19 case growth in the US. Nat Commun. (2021) 12:3118. 10.1038/s41467-021-23404-5 34035295 PMC8149701

[27] Courtemanche CJ Le AH Yelowitz A Zimmer R. School reopenings, mobility, and COVID-19 spread: evidence from Texas. Nat Bureau Econ Res. (2021). 10.3386/w28753

[28] HCDC. 8 loại vǎ′c-xin phòng COVID-19 đã đu'ọ'c câ′p phép tại Viẹ∧t Nam. HCDC (2021). Available online at: https://hcdc.vn/category/van-de-suc-khoe/covid19/tai-lieu-truyen-thong/8-loai-vacxin-phong-covid19-da-duoc-cap-phep-tai-viet-nam-27092973b1ad3fac17d53891de8b274f.html (accessed March 05, 2022).

[29] De Salazar PM Link NB Lamarca K Santillana M. High coverage COVID-19 mRNA vaccination rapidly controls SARS-CoV-2 transmission in long-term care facilities. Commun Med. (2021) 1:16. 10.1038/s43856-021-00015-1 35602197 PMC9053242

[30] Islam N Sharp SJ Chowell G Shabnam S Kawachi I Lacey B. Physical distancing interventions and incidence of coronavirus disease 2019: natural experiment in 149 countries. BMJ. (2020) 370:m2743. 10.1136/bmj.m2743 32669358 PMC7360923

[31] Lansiaux E Caut J-L Forget J Pébaý PP. Assessing the Efficiency of COVID-19 NPIs in France: A Retrospective Study Using a Novel Methodology. (2021). 10.21203/rs.3.rs-321360/v1

