# Supplementary material for: Movement restrictions, vaccine coverage, and reduction of the COVID-19 incidence rate in the fourth wave of the pandemic: Analysis results from 63 provinces in Vietnam
Source: Front Public Health. 2023 Jan 12;10:988107. doi: 10.3389/fpubh.2022.988107 (PMC9878390; doi:10.3389/fpubh.2022.988107)

Appendix

Figures 1−20 present the analysis results for each province, the north, central, south, and the whole country. Each figure includes four graphs denoted from a to d, which are explained as follows:

Figure 1−20a: The relationship between the daily rate of new COVID-19 cases and changes in mobility in some provinces in real time in public places (parks and residential areas, restaurants, etc.) was determined for each province according to the period from the median of the 50th case detection date to the end of the study period: early phase for the period before mean date and late phase for the time after mean day. Every time mobility restrictions were applied, mobility was suddenly high, especially in groceries and pharmacies, because people were worried and rushed to buy food and medicine. Daily new cases were extremely high, often peaking. This was observed in all provinces.

Figure 1−20b: Vaccination data were collected from May 3, 2021, to November 3, 2021, focused on two main vaccine types, AstraZeneca and Pfizer-BioNTech. After vaccination coverage increased, the number of deaths tended to decrease markedly.

Figure 1−20c: Relationship between the daily rate of new COVID-19 cases and changes in commuter travel across the country, in the north and south, and in each province. The period of measurement of change in mobility was from the date of detection of the 50th case in each province to November 3, 2021. The pandemic phase was determined for each province according to the average value of the 50th case date through November 3, 2021. The provinces that applied mobility restrictions included Ho Chi Minh City, Binh Duong, Dong Nai, Long An, Tien Giang, An Giang, Tay Ninh, Dong Thap, Khanh Hoa, Can Tho, Binh Thuan, Ba Ria, Soc Trang, Bac Lieu, Ba Ria, Bac Giang, Hanoi, and Northern, Central and Southern Vietnam. In the first stage of applying restrictions, the mobility value decreased, so the case ratio also decreased. The epidemic seemed to be under control at the end of the period, and the mobility value gradually increased, with the number of cases decreasing.

Figure 1−20d: An increase in mobility within residential areas, seen during most of the study period in all provinces, was associated with a decrease in COVID-19 rates but there was no additional effect in the early period and no association to any extent in the late period. During this period, the increase in movement within parks was associated with an increase in the incidence of the disease in provinces such as Ho Chi Minh City, Binh Duong, Dong Nai, Long An, Tien Giang, An Giang, Tay Ninh, Dong Thap, Khanh Hoa, Can Tho, Binh Thuan, Ba Ria, Soc Trang, Bac Lieu, Ba Ria, Bac Giang, Hanoi, and Northern, Central and Southern Vietnam. Movement around grocery stores and pharmacies was often associated with increased infection rates at the time of the onset of movement restrictions. There was also a correlation between case rates and mobility in most provinces.


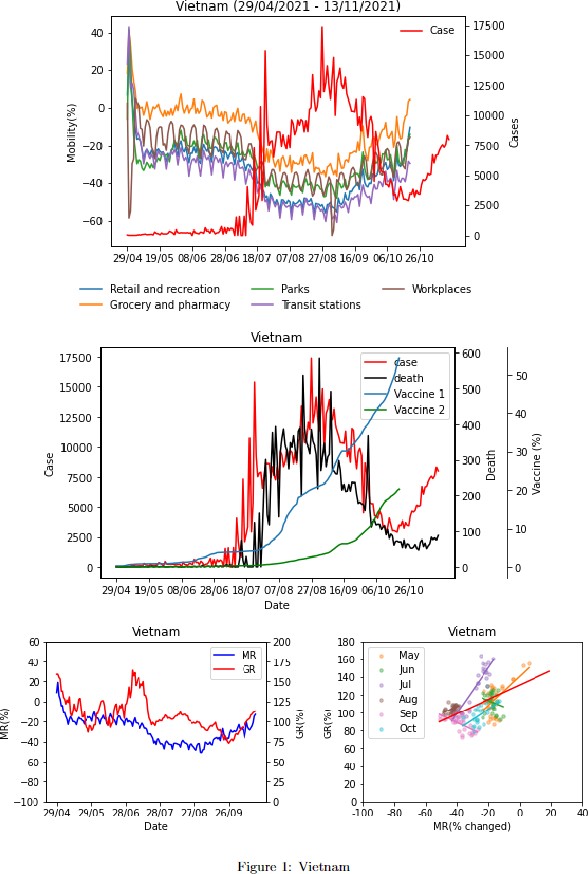


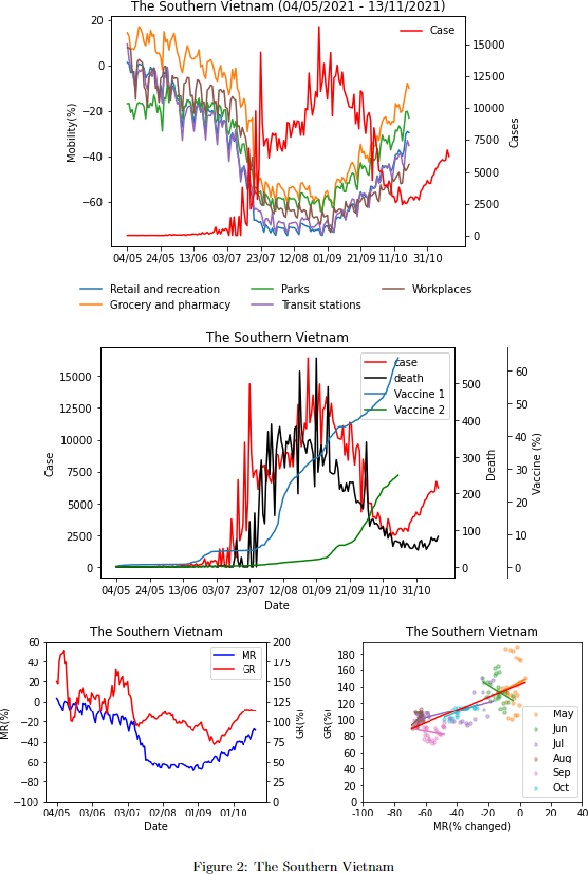


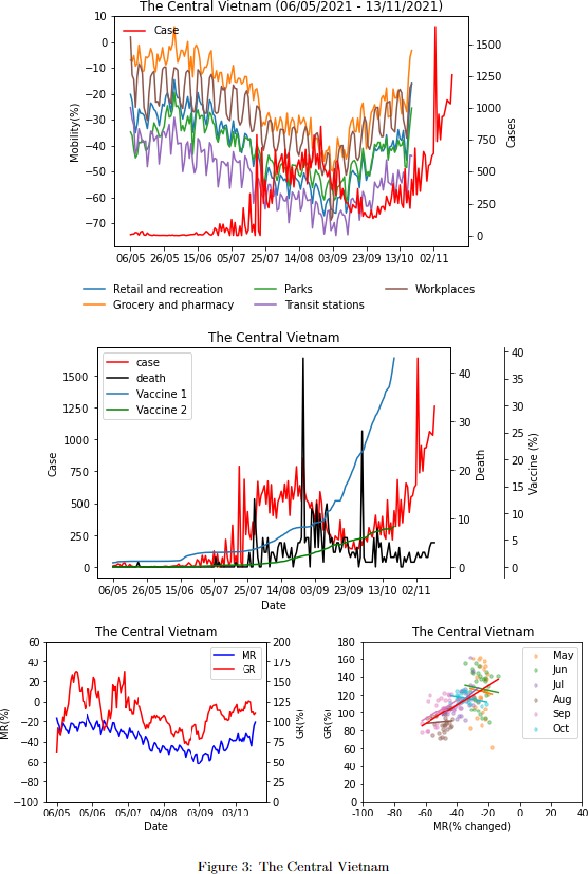


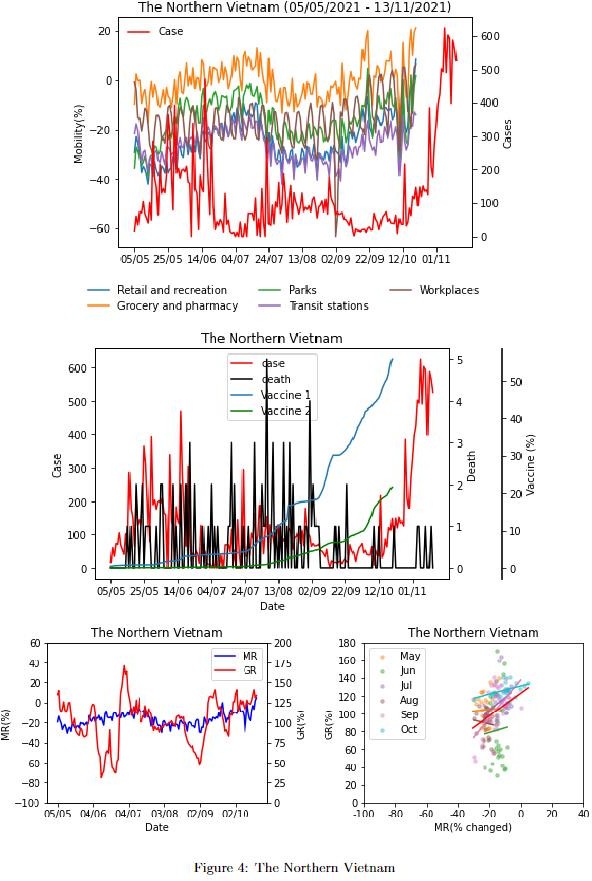


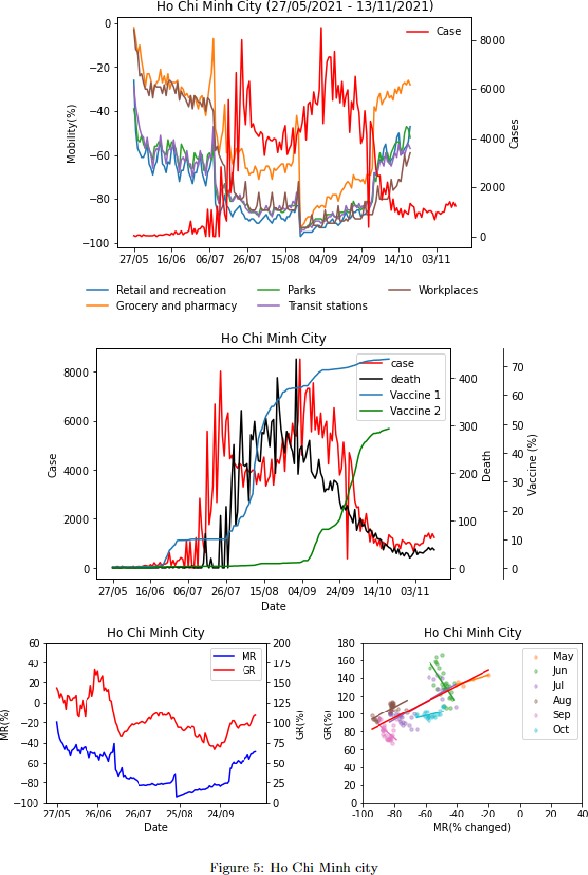


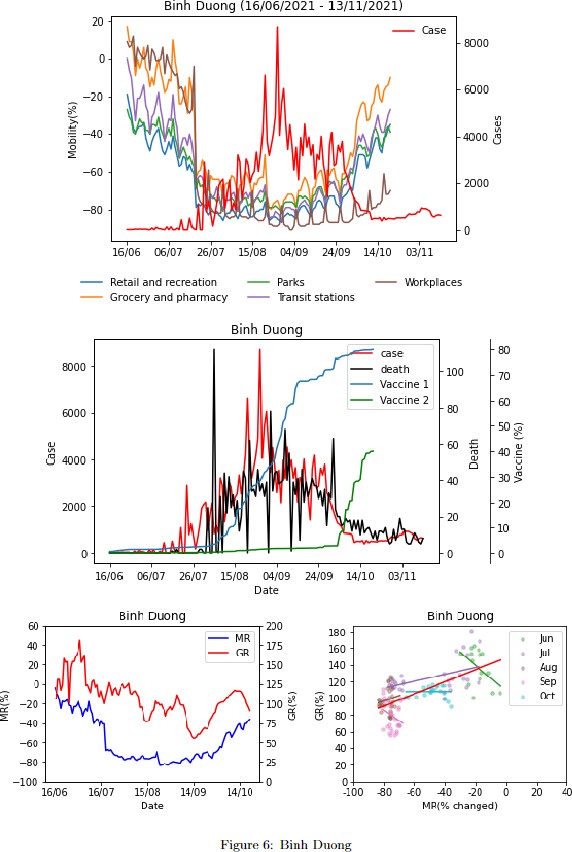


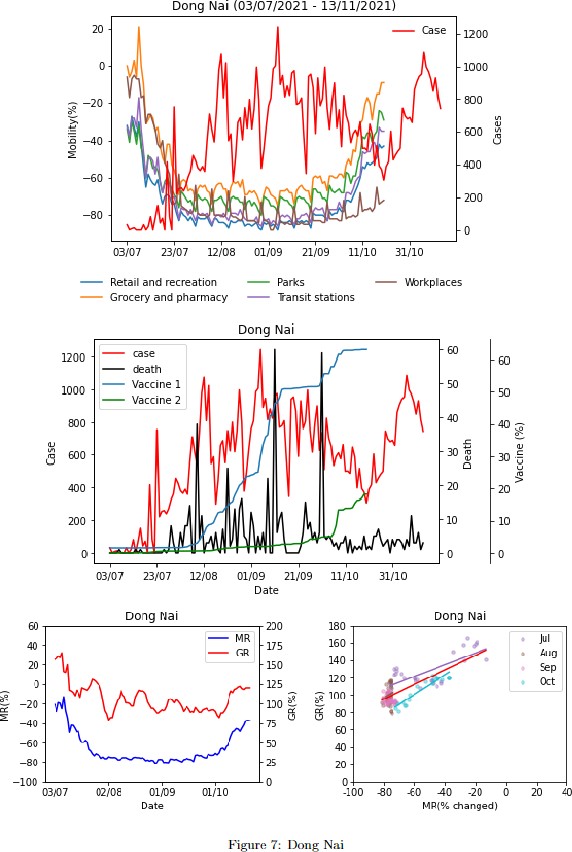


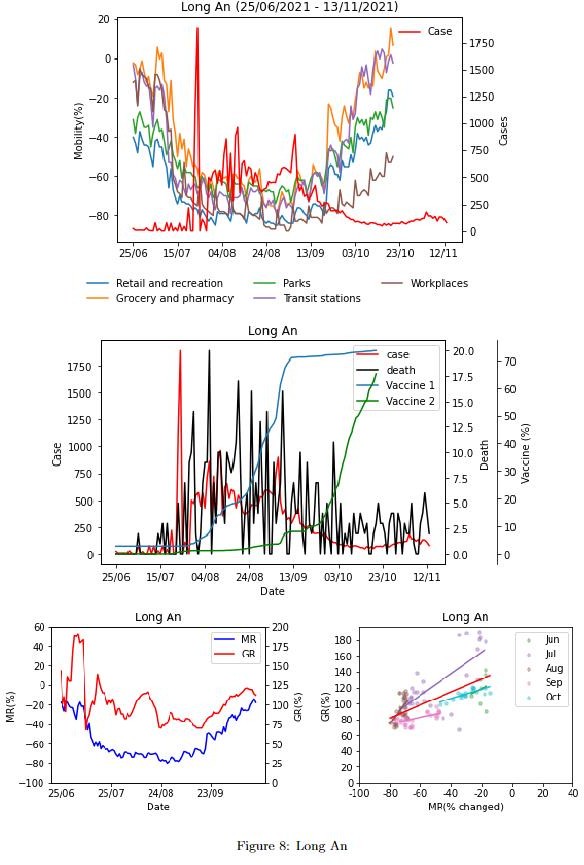


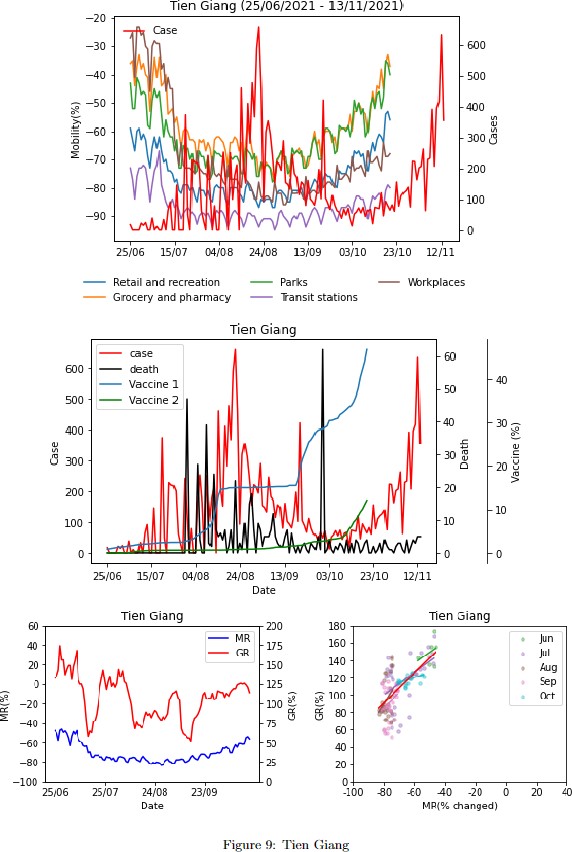


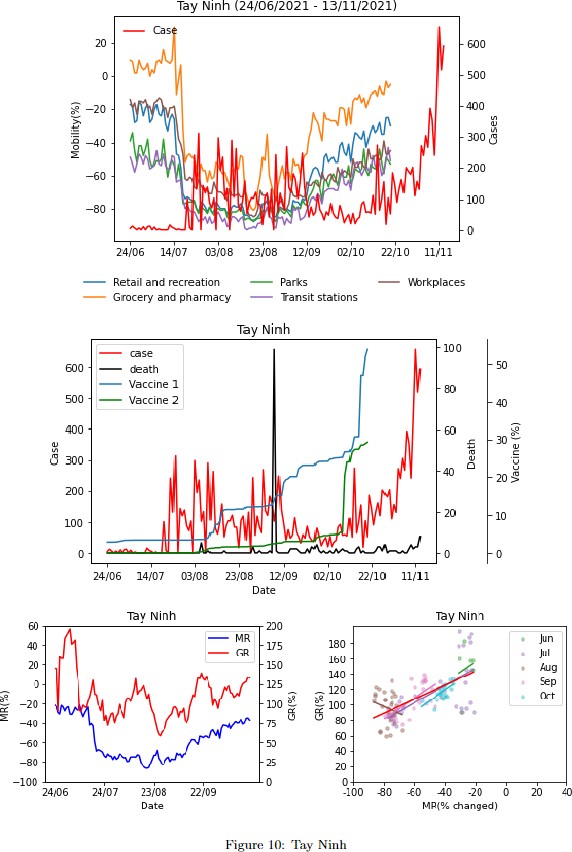


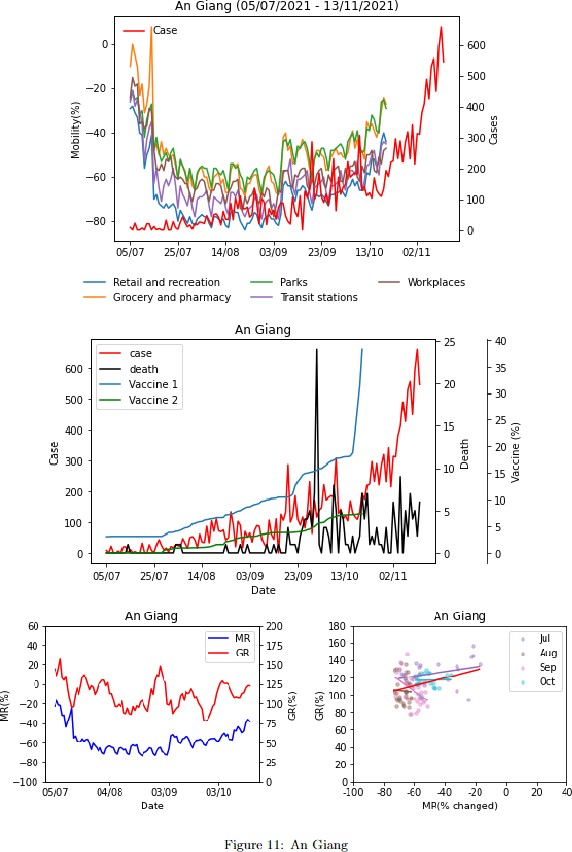


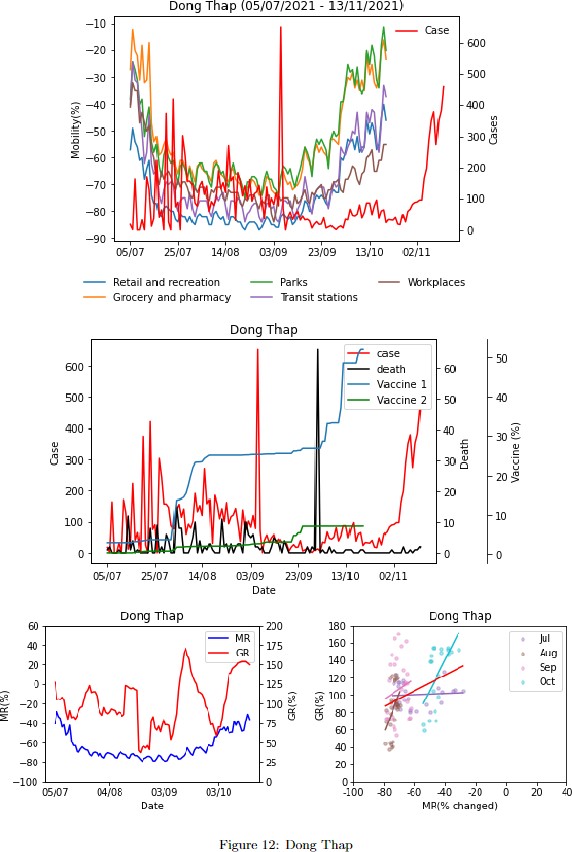


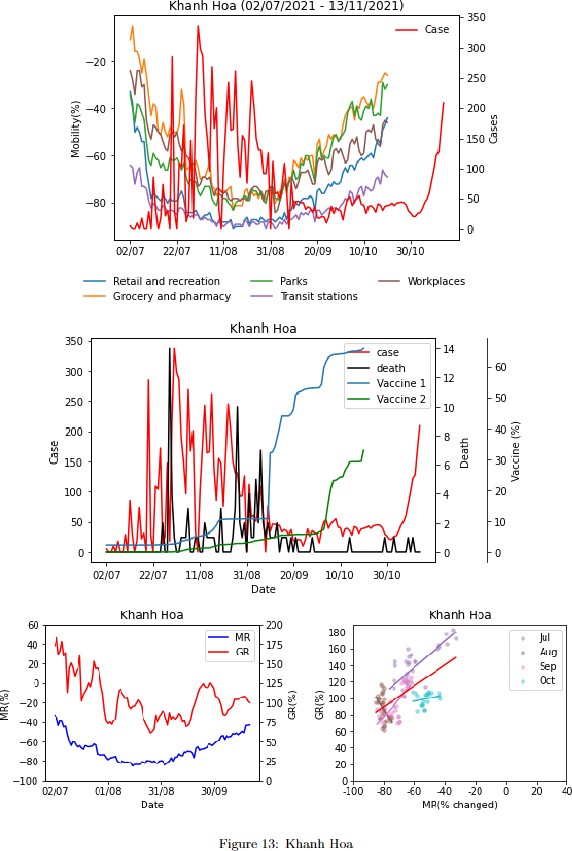


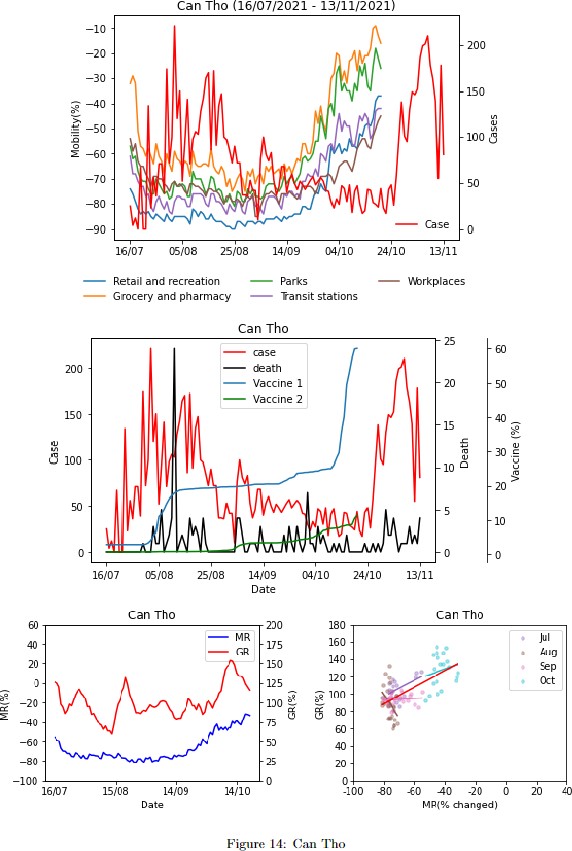


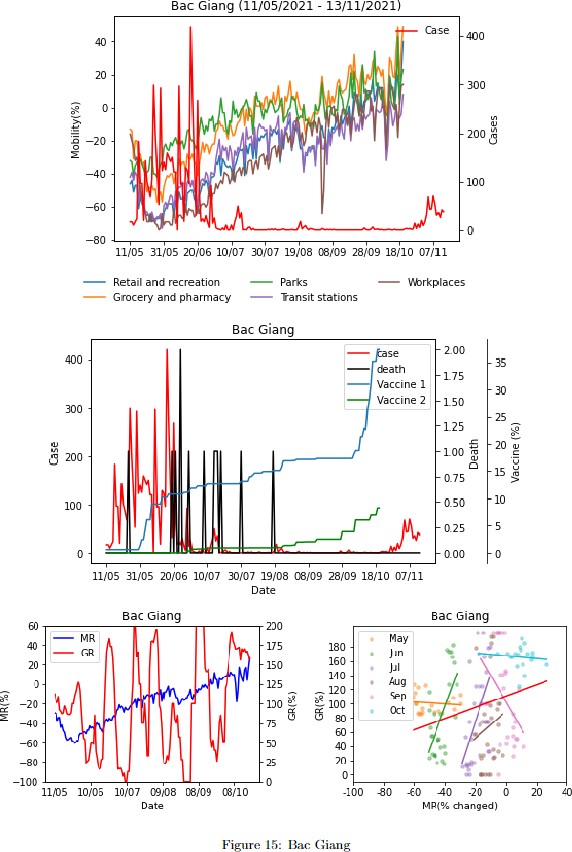


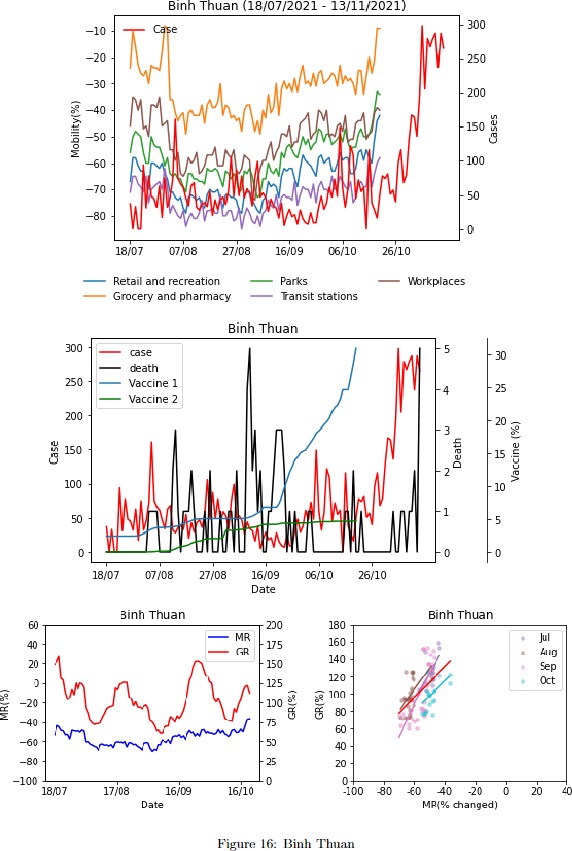


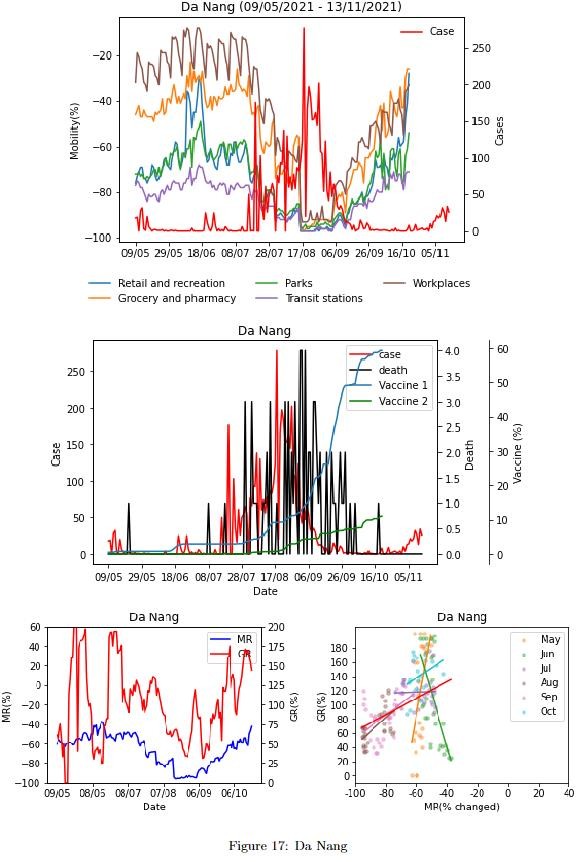


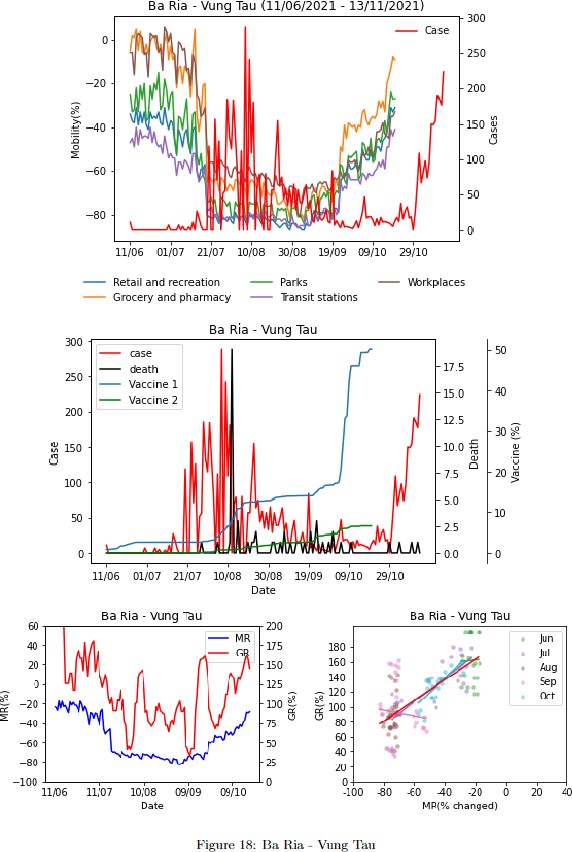


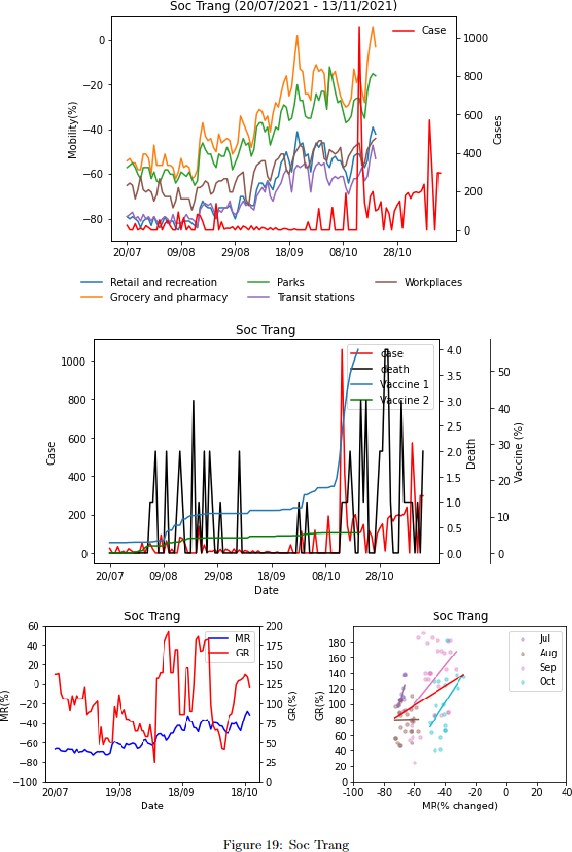


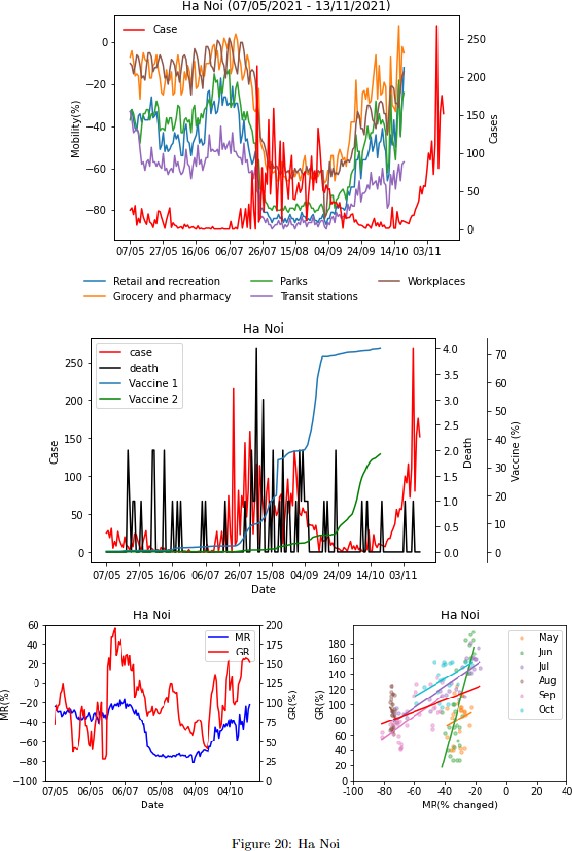

Supplement: Supplementary file 1 [file Data_Sheet_1.docx]
